# Fpr2/CXCL1/2 Controls Rapid Neutrophil Infiltration to Inhibit *Streptococcus agalactiae* Infection

**DOI:** 10.3389/fimmu.2021.786602

**Published:** 2021-11-24

**Authors:** Zeyu Sun, Wenhua Huang, Yuling Zheng, Peng Liu, Wenbo Yang, Zinan Guo, Decong Kong, Qingyu Lv, Xinyu Zhou, Zongmin Du, Hua Jiang, Yongqiang Jiang

**Affiliations:** ^1^ State Key Laboratory of Pathogen and Biosecurity, Institute of Microbiology and Epidemiology, Academy of Military Medical Sciences, Beijing, China; ^2^ Changchun University of Chinese Medicine, Changchun, China

**Keywords:** *Streptococcus agalactiae*, (GBS), formyl peptide receptor 2 (FPR2), CXCL1 = chemokine (CXC) ligand 1, chemokine (C-X-C motif) ligand 2, neutrophil (PMN)

## Abstract

*Streptococcus agalactiae*, also known as group B streptococcus (GBS), can cause pneumonia, meningitis, and bacteremia, making it a pathogen that can increase the risk of death in newborns and immunodeficient individuals. Neutrophils are the first barrier to a host’s innate immune defense against these infections. Fpr2(Formyl peptide receptor 2) is an important chemotactic receptor of neutrophils, though its activation would cause pro- and anti-inflammatory effects. In this study, we found that mice without Fpr2 receptor were highly susceptible to GBS infections. These mice demonstrated decreased chemotaxis to neutrophils, decreased bactericidal ability of neutrophils, and high mortality. RNA-seq and Luminex assay indicated that Fpr2 activates key signal molecules downstream and produces chemokines CXCL1/2 to chemotaxis neutrophils. Like Fpr2^-/-^, CXCL1/2 or neutrophil depletion impairs host’s ability to defend against GBS infection. Altogether, these data indicate that Fpr2 contributes to a host’s ability to control GBS infection and that a lack of Fpr2 was associated with selective impairment during the production of chemokines CXCL1 and CXCL2 as well as neutrophil recruitment. Here, We clarified that Fpr2, as a chemotactic receptor, could not only directly chemotactic neutrophils, but also regulate the production of chemokines to control infection by chemotactic neutrophils.

## Introduction

Streptococcus agalactiae (GBS), is an encapsulated Gram-positive bacterium that colonizes the human urogenital tract, often asymptomatically in healthy adults (1). GBS can cause severe infections, including pneumonia, sepsis, and meningitis, and is a major cause of infection-based mortality in neonatal infants and elderly or immunocompromised adults, such as liver cirrhosis, diabetes, and other malignancies (2, 3). Innate immunity plays an important role in a host’s resistance to these bacterial infections. Neutrophils and macrophages are important components of innate immunity: they engulf bacteria and produce ROS, antimicrobial peptides, and other substances that can kill bacteria. Neutrophils represent the first line of defense during infections and represent approximately 50% to 70% of the circulating human leukocytes (4). Early, rapid, and accurate chemotaxis of neutrophils to the infection site is important to fight off severe infections. The extravasation process is induced by different chemoattractants, which activate neutrophils *via* G-protein-coupled seven-transmembrane cell surface receptors(GPCR). Early autopsies of newborn patients with pneumonia caused by GBS infections demonstrated that a large number of bacteria were accumulated in the alveoli, though neutrophils could not effectively migrate to the alveoli and was primarily concentrated in capillaries. Neutrophils treatment with extracted type III GBS antigens can reduce the chemotaxis of platelet-activating factors (PAF), leukotriene B4 (LTB4), and fMLP (formylmethionyl leucyl phenylalanine) to neutrophils. The inhibitory effects on the fMLP chemotaxis does not act by reducing the binding of fMLP to the receptor (5), making it possible to terminate the downstream chemotactic reaction of Fprs, or reduce the chemotactic substances produced by Fprs and etc. Any of these reactions would affect the chemotaxis of neutrophils.

Fprs belong to the classical chemotactic GPCR subfamily, and they are expressed in a wide variety of cell types, including neutrophils and macrophages. In humans, there are three FPRs: FPR1, FPR2, and FPR3. Sequence comparison of receptors revealed a similarity of 70%. Mouse Fpr (*mFpr*) gene family consists of at least 8 members (6, 7), while the mFpr1/2 is the orthologue of human FPR1/2. The activation of FPR1 and FPR2 can cause a series of signal transduction events, resulting in myeloid cell migration, mediator release, enhanced phagocytosis, transcription of new genes, involvement in host responses to bacterial infection, tissue repair, and wound healing (7). Fpr1/2 is an important component of the innate immune response to defend against bacterial infections (8). Studies assessing *Listeria monocytogenes*, *Staphylococcus aureus*, and *Escherichia coli*, found that both Fpr1/2 activation and inflammation were needed for the host to control bacteria. However, for *Streptococcus suis* and *Streptococcus pneumonia*, Fpr1/2 activation aggravated inflammatory damage in the host.

The ligand of Fpr1 is primarily N-formyl peptides produced in nature by the degradation of either bacterial or host cell mitochondrial proteins, which directs neutrophils to the bacteria or damaged tissue and shows a pro-inflammatory response. However, Fpr2 interacts with a variety of structurally diverse pro-inflammatory and anti-inflammatory ligands, including biomolecules from pathogenic bacteria (such as *L. monocytogenes* fMIVIL and *E. coli* fMLF), mammalian hosts (such as SAA, LXA4) and some synthetic polypeptides (such as WKRMVM, MMK-1, *etc)*. In a review of Fpr function in *J Autoimmun* from 2017, the author summarized the function of Fpr very well. Among them, the main role of Fprs in pathogenic infection is Fpr1/Fpr2 mediated neutrophil recruitment in bacterial infection, that is, in *L. monocytogenes* infection, Fpr1/2 directly chemoattract neutrophils through the receptor itself before CXCL1/2 is produced. In Tollip-deficient mice DSS-induced colitis and acute sepsis models, neutrophils expression Fpr2 is reduced, reducing neutrophils chemotaxis. The ability of Fpr2 to mediate pro-inflammatory and anti-inflammatory biological effects could be due to different receptor domains used by different agonists, leading to different signal transductions. Meanwhile, Cooray et al. found that Fpr2 transmitting both pro-and anti-inflammatory signals is related to whether it can be homodimerized after binding to the ligand (9).

Compared with Fpr1, Fpr2 displayed diverse ligands, different signal pathways in different cells, and complex functions after activation. Therefore, we established a bacteremia model of GBS infection to evaluate the effect of Fpr2 on host’s chemotaxis on neutrophils and neutrophils bactericidal functioning. For GBS infections, Fpr2 does not chemotactic neutrophils directly through the receptor itself, but through the activation of the receptor to produce chemokine CXCL1/2. The absence of Fpr2 damages many bactericidal function of neutrophils, decreasing the survival rate. In conclusion, Fpr2 is very important for the host to defend against GBS infection.

## Materials and Methods

### Bacterial Strains

The GBS strain BJCH_SA4 used here is a high virulent type III strain which is stored in our lab. The bacteria were grown overnight in Todd–Hewitt broth (THB) at 37°C containing 5% CO_2_, washed twice in nonpyrogenic PBS after centrifugation (PBS; 0.01 M phosphate, 0.15 M NaCl, pH 7.2), and resuspended to the desired concentration. Heat-killed bacteria (HK-GBS) was prepared according to the methods previously reported (10). In each experiment, the actual number of injected bacteria was determined by colony counts. In βH/C-blocking experiments, the inhibitor DPPC(dipalmitoyl phosphatidylcholine, sigma-adlrich, USA) was suspended in PBS by sonication and added to GBS for 10 min before being injected into the orbita vein.

### Mice

Fpr2^-/-^(Fpr2 knockout [KO]) mice were constructed from Cyagen Biosciences (Guangzhou, China) according to methods previously reported (11). The control, wild-type (WT) C57BL/6 mice (6-8 weeks old), were purchased from Charles River (Beijing, China). The mice were maintained and bred under Specific pathogen-free(SPF) conditions in the animal facilities of the Academy of Military Medical Sciences.

### Experimental Models of GBS Disease

Six-week-old mice were injected intraperitoneally (*i.p.*) or intravenously (*i.v.*) with the BJCH_SA4 GBS strain. For the two GBS-induced infected models, mice were injected *i.p.* with 10^8^CFU/ml mid-logarithmic-phase GBS or mice were firstly anesthetized and then injected in the orbital vein with 10^8^CFU/ml mid-logarithmic-phase GBS and monitored daily for survival. For receptor inhibition experiment, WT mice were treated with Boc-2 (600ng/kg) or vehicle for 1 h before GBS infection. Boc-2 peptide together with the lipidated peptidomimetic Lau-(Lys-βNSpe)6-NH2 ligand, were recently found to be selective inhibitors for Fpr2. For neutrophils depletion experiment, WT mice were pretreated *i.p.* with 100μg of rat monoclonal anti-mouse Ly6G antibodies or rat IgG2a isotype control (BD Pharmingen, USA) 24 h before *i.v.* inoculation with 2×10^8^ CFU/ml of GBS. For Chemokine depletion experiment, the mice were injected *i.p.* 3h before the GBS challenge with 100 μg rat monoclonal antibodies (mAbs) specific for CXCL1, CXCL2, or IgG2a and IgG2b isotype controls (R&D, Minneapolis, MN, USA). For monocyte/macrophage depletion experiment, mice were pretreated *i.p.* with 200μg of clodronate liposome or control PBS liposomes (Yeasen Biotech Co. Ltd, China) 24 h before *i.v.* inoculation with 2×10^8^ CFU/ml of GBS. After the infection, the peripheral blood was taken from the orbital vein into an EDTA anticoagulation tube for follow-up experiments. The organs were placed in preweighed sterile tubes containing PBS and homogenized in a gentle tissue disruptor.

Meanwhile, the HK-GBS was injected *i.p.* at a dose of 0.5 mg/mouse in 0.2 ml of PBS, while the peritoneal lavage fluid was collected at predetermined times to measure cell numbers *via* flow cytometry and cytokine concentrations using ELISA.

### Cytokine Measurement and Luminex Assay

Concentrations of cytokine in the plasma and supernatants were detected by multiplexed Luminex xMAP assay (Cytokine & Chemokine 36-Plex Mouse ProcartaPlex™ Panel 1A, Thermo Scientific, USA) or ELISA according to the manufacturer’s instructions.

### Flow Cytometry

After the mice were infected for 3 hours and 6 hours, the peripheral blood was taken into an EDTA anticoagulation tube. The red blood cell was lysed on ice for three minutes and centrifuged at 2300 rpm for 3 minutes, while the supernatant was removed to obtain the cells. The same method was used to obtain cells from the peritoneal lavage fluid. The absolute count of blood leukocytes in the blood and peritoneal lavage fluid samples were determined using a BD TruCount system (BD Biosciences, USA). The cells were stained with PE anti-mouse Ly6G, PerCP/Cyanine5.5 anti-mouse CD45, FITC-anti-mouse CD11b, Apc anti-mouse Ly6C, PE/cy7 anti-mouse CD19, and Apc anti-mouse CD3. Antibodies were purchased from Biolegend (San Diego, CA, USA). All of the above-stained cells were assayed using a BD FACSVerse flow cytometer and data were analyzed using FlowJo software.

### RNA-Seq and Bioinformatic Analysis

After three hours of GBS infection, the WT and Fpr2 knockout mice were used to isolate neutrophils with the Tianjin Haoyang Neutrophil Kit. Once the purity exceeded 95%, trizol was added, and the samples were placed in liquid nitrogen and sent to the Beijing Novogene company, which conducted sequencing and analyzed the results. The reads of each gene were counted by feature counts, and the differentially expressed genes had adjusted p-values <0.01 and were differentially expressed by at least 2-fold. Quantitative PCR (qPCR) was also performed to verify the results of RNA-seq. The SYBR Green PCR method was used according to the instructions of the manufacturer of the PowerUp™ SYBR™ Green Master Mix (Thermo Scientific, USA), while the GAPDH gene was used as an internal control.

### Multiplex Immunofluorescence (mIF) Analyses

Liver FFPE tissue of mice was used as an experimental sample. All tissues were cut into 4-μm thick sections. The slides were deparaffinized in xylene for 30 mins and rehydrated in absolute ethyl alcohol for 5 mins (twice), 95% ethyl alcohol for 5 mins, 75% ethyl alcohol for 2 mins. The slides were washed with distilled water three times. A microwave oven was used for heat-induced epitope retrieval; during epitope retrieval, the slides were immersed in boiling H_2_O_2_ buffer (PH9.0; Histova Biotechnology, China) for 15 mins. Primary antibodies were performed as follows: Ly6G (1:500, 87048s, Cell Signaling Technology, Danvers MA, USA), CXCL1 (1:500, ab269939, Abcam, USA). All primary antibodies were incubated for 1 h at 37°C. The slides were then incubated with Opal Polymer HRP Ms+Rb (2414515; PerkinElmer, Massachusetts, USA) for 10 min at 37°C. An Opal Seven-Color IHC Kit (NEL797B001KT; PerkinElmer, Massachusetts, USA) was used for visualization. After each cycle of staining, heat-induced epitope retrieval was performed to remove all antibodies, including primary antibodies and the Opal Polymer HRP Ms+Rb. The slides were counterstained with DAPI for 5 mins and enclosed in Antifade Mounting Medium (I0052; NobleRyder, Beijing, China). The slides were scanned by multi-spectral technology [Vectra 3.0(TM)/Polaris(TM)], and analyzed by Inform software.

### Reagent Information

U73122(Catalog Number: HY-13419), PP1(Catalog Number: HY-13804), Wortmannin (Catalog Number: HY-10197) and Gallein(Catalog Number: HY-D0254) were purchased from MCE (USA). PLC specific inhibitor U73122 at 50μM, Src-specific inhibitor PP1 at 20μM,PI3K-specific inhibitor Wortmannin at 50μM, Gβγ-specific inhibitor Gallein at 20μM.

### Histopathological and Scoring

Mice were infected and subsequently euthanized, after which the liver, lung and spleen tissues were recovered and fixed in 10% buffered formalin. These tissues were stained with hematoxylin and eosin and examined *via* OlympusBX53 microscopy. The histological assessment was scored as previously described (12).

### MPO and Nets Measurement in Blood

Peripheral blood was taken from the orbital vein 3h after infection. Myeloperoxidase (MPO) protein level was determined using a MPO Assay Kit (Mlbio, Shanghai, China) according to the manufacturer’s instructions. We isolated neutrophils and adjusted the number of neutrophils in WT and Fpr2 group to be the same, then stimulated with GBS (MOI 10:1). Nets protein level was determined using a Nets Assay Kit (Mlbio, Shanghai, China) according to the manufacturer’s instructions.

### Measurement of ROS Production

We isolated neutrophils and adjusted the number of neutrophils in WT and Fpr2 group to be the same, then stimulated with GBS(MOI 10:1), then DCFH-DA was performed for labeling and the cells were detected using the FITC channel in upstream mode. The kit was purchased from China Biyuntian Biological Company.

### GBS Killing by Blood

Mouse peripheral blood was incubated with 2×10^6^ CFU/ml GBS for 1h at 37°C. 500ul of saponing was added, after which the cells were lysed on ice for 5min. 100ul serial dilutions were prepared and spread on THB plate and incubated overnight at 37°C for the colony count.

### Ethics Statement

This research was performed in compliance with the guidelines of laboratory animal care and was approved in China. All experimental procedures were approved by the Animal Ethics Committee of the Academy of Military Medical Sciences.

### Statistical Analysis

All data were analyzed with GraphPad Prism (version 7.0, GraphPad Software, La Jolla, CA, USA). The differences between groups were calculated using an unpaired t-test and a log-rank test, respectively. Data are shown as means ± SD. Differences were considered statistically significant when *P <*0.05: *P < 0.05, **P < 0.01, and ***P < 0.001.

## Result

### Fpr2^-/-^ Mice Are Highly Susceptible to GBS Infection

Fpr2 receptors can play a pro-inflammatory or anti-inflammatory role when it is activated. To examine the ability of Fpr2 to mediate host responses to GBS infection, live bacteria were injected *i.v.* and qPCR was detected in the peripheral blood. We found that Fpr2 expression levels were the highest (**Supplementary Figure 1A**). Then Fpr2 receptor was inhibited with Boc-2 and infected with GBS *i.v.* and we found that the mortality rate was higher than that of WT mice (**Supplementary Figure 1B**).

**Figure 1:**
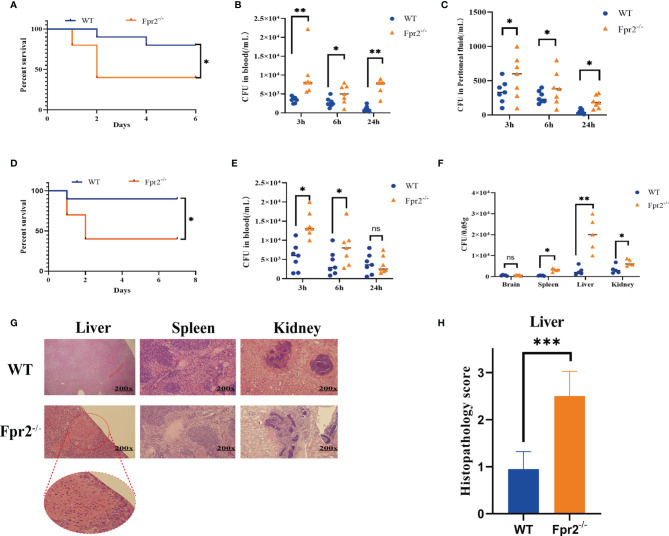
Mice lacking Fpr2 are highly susceptible to GBS infection. **(A)** Survival of WT or Fpr2-deficient mice after *i.p.* challenge with 2×10^8^ CFU/ml of GBS; each included 10 animals per group. The survival rates of the two groups were compared using a log-rank test. **(B**, **C)** Numbers of bacterial in peripheral blood and peritoneal lavage fluid of WT or Fpr2-deficient mice at 3h, 6h, 24h after *i.p.* challenge with 2×10^8^ CFU/ml of GBS. Each was conducted on a different animal over the course of two experiments, each of which included 6-8 animals per group. **(D)** Survival of WT or Fpr2-deficient mice after *i.v.* challenge with 2×10^8^ CFU/ml of GBS, each of which involved 10 animals per group. The survival rates of the two groups were compared using a log-rank test. **(E)** Numbers of bacterial in peripheral blood of WT or Fpr2-deficient mice at 3h, 6h, 24h after *i.v.* challenge with 2×10^8^ CFU/ml of GBS. Each was conducted on a different animal, with 6-8 animals per group. **(F)** Numbers of bacterial in brain, spleen, liver and kidney of WT or Fpr2^-/-^ mice at 24h. **(G)** Hematoxylin and eosin staining of infected tissue sections at 24 h of infection. The horizontal line indicates 200 microns. **(H)** Clinical scoring was performed in accordance with a formerly developed scoring list for a bacterial mouse model. Data are shown as mean ± SD. *P < 0.05, **P < 0.01, ***P < 0.001, ns means non sense.

During the experiment, we have found in the preliminary experiments that when the mice are not infected with GBS, the organs of the Fpr2 knockout mice are not damaged, and the mice themselves do not have any defects or injuries. To further confirm the role of Fpr2 in GBS infections, we prepared Fpr2^-/-^ mice and infected GBS by *i.p.* Our results demonstrated that the survival rate of mice in the Fpr2^-/-^ group was less than 50% at 48h after infection, which was significantly lower than that in the WT group (*P*<0.05, **Figure 1A**). Meanwhile, we obtained bacterial titers from peritoneal lavage fluid and blood samples from a separate set of Fpr2^-/-^ or WT mice. At 3h, 6h, and 24h, the number of bacteria in the peritoneal lavage fluid and blood in the Fpr2^-/-^ group was significantly higher than that in WT mice (*P*<0.05, **Figures 1B, C**). These results demonstrated that the loss of an Fpr2 receptor decreased the host’s ability to control GBS infections.

Furthermore, we used intravenous injections to observe the clearance of GBS in blood and the invasion of GBS into the organs. Our results demonstrated that 48 h after infection, the survival rate of mice in the Fpr2^-/-^ group decreased significantly, to less than 50%. However, only one mouse in the WT group died at 24 h, while the other mice survived for more than seven days (**Figure 1D**). The results of the colony count indicated that GBS levels in the peripheral blood, spleen, liver, and kidney of Fpr2-/- mice were significantly higher than in WT mice after 24h (**Figure 1E, F**). However, we found no difference in the number of colonies between the two groups and no symptoms of meningitis in the brain tissue, which was repeated three times. This might be related to the short observation time.

There are several reports on liver damage caused by Fpr2 deletion (13–15). In this study, we analyzed pathological damage to the liver, spleen, and kidney. Our pathological analysis demonstrated that during GBS infections, Fpr2 loss damaged the spleen, and liver though damage to the liver was the most serious (**Figure 1G**). Briefly, the pathological analysis of liver showed pathological changes characteristic of severe tissue destruction, and large areas of piecemeal necrosis following GBS infection in Fpr2^-/-^ mice. No obvious pathological alterations in liver were found following GBS infection in WT group. For kidney, there was no glomerular, interstitial or vascular injury in the WT group, but these pathology characteristics became readily observable in the Fpr2^-/-^ group. Cellular proliferation and/or membrane thickness in glomeruli, and interstitial inflammatory cell infiltration were observed. For spleen, there was little difference between the two groups (**Figure 1G**). Fpr2^-/-^ liver pathological injuries were determined using a semi-quantitative score (**Table 2**), and we found a significant difference in the pathological injury score for Fpr2^-/-^ (*P*< 0.001, **Figure 1H**). These results are consistent with those of the colony counts.

Altogether, our results conclusively indicated that Fpr2^-/-^ mice were more susceptible to GBS infections and could not control the invasion of GBS from the blood to the organs. This confirms that Fpr2 is an important receptor for hosts and is needed to resist GBS infections.

### Impaired Neutrophil Recruitment in Fpr2-Deficient Mice During GBS Infections

**Figure 2:**
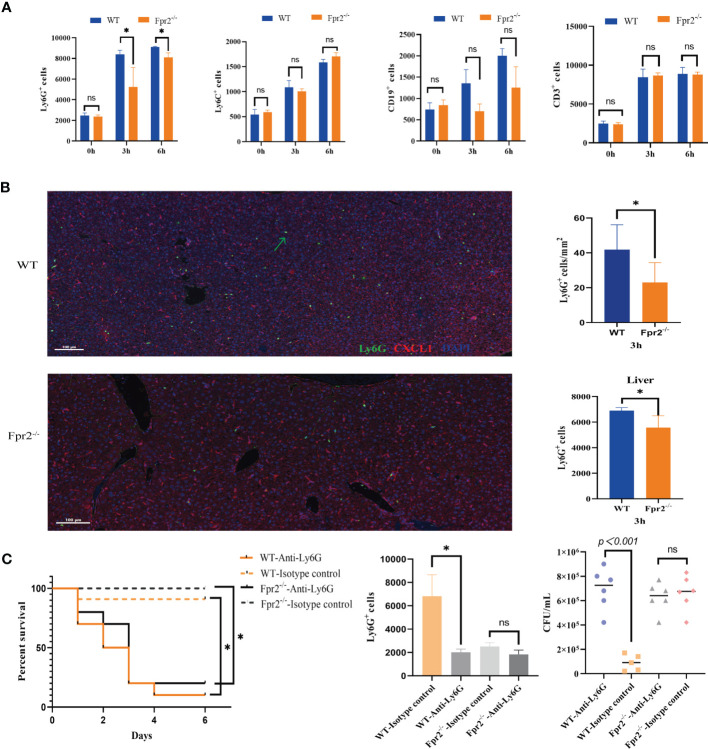
Impaired neutrophil recruitment in Fpr2-deficient mice during GBS-infection. **(A)** Cell counts in peripheral blood samples after *i.v.* challenge with GBS at 0h, 3h, and 6h; kinetics of recruitment of cells positive for CD11b, Ly6G(neutrophils), Ly6C(monocyte/macrophages), CD19 (B lymphocytes) and CD3 (T lymphocytes) in WT and Fpr2-defective mice. **(B)** Representative images of liver tissue sections labelled with mIHC(Left), neutrophil number analyzed by inform software (Right Up) and Flow cytometry (Right down). Three-color, multiplex immunofluorescent images of mice liver tissue sections displaying the spatial distribution of different immune lineages and markers. The technique labels three channels as follows: Ly6G (green), CXCL1(red)and DAPI (blue). **(C)** Survival and numbers of bacterial in blood of WT and Fpr2^–/–^ mice pretreated with anti-Ly-6G antibody or the isotype control after *i.v.* challenge with 2×10^8^ CFU/ml of GBS. Data are representative of three independent experiments (n = 5 mice in the per group) and are shown as mean ± SD. *P < 0.05, ns means non sense.

We next sought to analyze the mechanisms underlying increased susceptibility to GBS infections in the absence of Fpr2. Because the Fpr2 receptor is chemotactic, we hypothesized that Fpr2^-/-^ mice reduced inflammatory cell recruitment at GBS infection sites. As seen from the liver pathological sections of GBS infections, the number of inflammatory cells in the liver tissue of Fpr2^-/-^ mice is significantly lower than in WT mice. To determine the type of reduced inflammatory cells, we firstly determined the number and types of cells present under basal conditions in the peripheral blood and found that they did not differ between Fpr2^-/-^ mice and WT mice (data not shown). The mice were then infected *i.v.* with GBS, after which the peripheral blood was obtained at predetermined times. Compared to monocytes/macrophages, B cells, and T cells, the recruitment of neutrophils in Fpr2^-/-^ mice significantly blunted in early stages 3 and 6 h after the infection (**Figure 2A**). We observed that damage to the liver is most serious after the GBS infection, indicating that neutrophils in the liver suffer similarly. Therefore, we used mIF to detect Ly6G^+^ cells in the liver and found a decrease in the number of neutrophils in the liver 3h after infection. Flow cytometry also demonstrated significant lower number of neutrophils in the liver of Fpr2^-/-^ mice (**Figure 2B**).

Data above suggested that the over-sensitivity to GBS infections seen in Fpr2^-/-^mice is related to its reduced ability to recruit neutrophils to the infection site. Our results for GBS infection showed that a 80-90% mortality rate in both that were treated with anti-Ly6G (assessed 96 hours after GBS infection). Under the same experimental conditions, the mortality rate of all control animals was only ~20% (**Figure 2C**). Thus, our data supporting that mice lacking neutrophils are unable to control the growth of GBS, and Fpr2^-/-^ decreased host resistance to GBS infections, which is related to the impaired increase in neutrophils.

### Low Neutrophils in Fpr2^-/-^ Mice Was Unrelated to GBS Cytotoxicity

**Figure 3:**
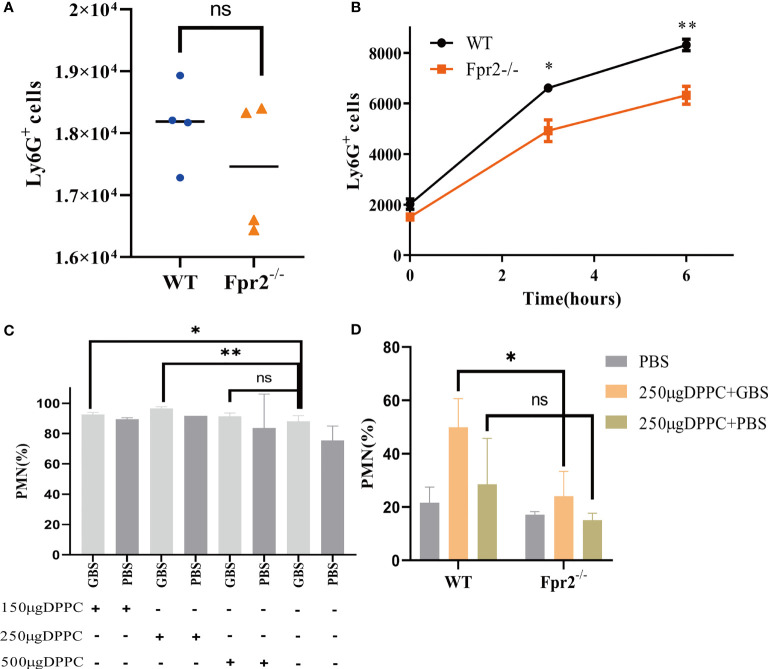
GBS has a slight impact on the number of neutrophils. The number of neutrophils in peripheral blood **(A)** and peritoneal lavage fluid **(B)** from WT and Fpr2^-/-^ mice, sampled 3h after *i.p.* injection of heat-killed GBS (0.5 mg/mouse). **(C)** The blocking effects evaluation of three doses of DPPC on neutrophils (% of CD45^+^ cells) after GBS-infection in WT mice. **(D)** Blocked by DPPC, difference in the number of neutrophils (% of CD45^+^ cells) between WT and Fpr2^-/-^ groups observed after injecting the GBS. Data are representative of three independent experiments with n = 5 mice per group. Data are shown as mean ± SD. *P < 0.05, **P < 0.01, ns means non sense.

While we observed fewer neutrophils in Fpr2-knockout mice than in WT mice, it is unclear whether neutrophils levels are associated with low chemotaxis or the high number of living bacteria in Fpr2^-/-^ mice. The cytotoxin βH/C is an important virulence factor produced by GBS and can form pores on the surface of eukaryotic cells and lyse neutrophils (16). Does neutrophils impairment increase in Fpr2^-/-^ mice due to the high concentration of βH/C in the blood? To exclude this possibility, we used HK-GBS to avoid βH/C interference. The mice were injected with HK-GBS *i.p.* and 3h later, blood and the peritoneal lavage fluid were obtained to count the neutrophils number. Our results demonstrated that there was no significant difference in the chemotaxis of neutrophils in the blood, while neutrophils levels in Fpr2^-/-^ mice are significantly less than that in the WT group in the peritoneal lavage fluid (**Figures 3A, B**).

Thermal inactivation can affect the virulence of bacteria, leading us to block GBS with the βH/C inhibitor DPPC (16), which could block the cytotoxicity of βH/C (**Supplementary Figures 1E, F**) and perform an intravenous injection to observe the difference in neutrophils. After confirming that 250µg/mouse DPPC can effectively block the cytotoxicity of βH/C (**Figure 3C**), 2×10^8^ CFU/ml GBS was incubated with DPPC for 10 min and injected into mice *i.v.* Our results demonstrated that after eliminating βH/C interference, neutrophils levels were still reduced in the absence of Fpr2 (**Figure 3D**). The results of HK-GBS and DPPC blocking βH/C demonstrated that GBS slightly impacts neutrophils levels, but is not the primary reason.

### Absence of the Fpr2 Receptor Affects CXCL1/2 Production

**Figure 4:**
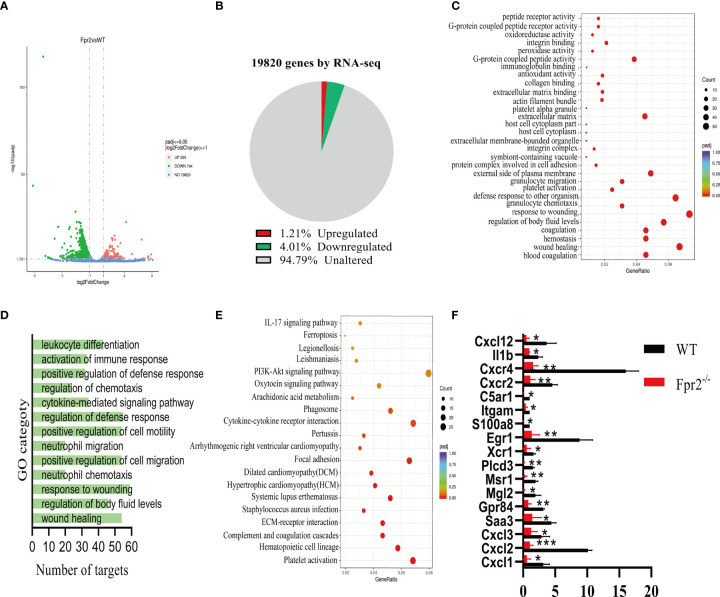
RNA-seq analysis of Fpr2-modulated genes. **(A)** A volcano plot illustrating differentially regulated gene expression from RNA-seq analysis between WT and Fpr2^-/-^. Genes upregulated and downregulated are shown in red and green, respectively. Values are presented as the log2 of tag counts. **(B)** RNA-seq comparison revealed a total of 1033 genes expressed, of which 239 genes were upregulated and 794 genes were downregulated. **(C)** KEGG pathway analysis of downregulated targets in Fpr2-deficient transcriptome. **(D, E)** Gene ontology (GO) functional clustering of genes that were downregulated for biological processes. **(F)** qPCR validation analysis of the indicated genes regulated by Fpr2. Data are shown as mean ± SD. *P < 0.05, **P < 0.01, ***P < 0.001.

The GBS infection of Fpr2^-/-^ mice will reduce the ability of neutrophils to recruit to the infected site, leading us to assess the factors that affect the chemotaxis of neutrophils. RNA-seq analysis was performed on neutrophils in Fpr2^-/-^ and WT mice infected with GBS. Of 1033 significantly differentially expressed genes (DEGs) in Fpr2^-/-^, the transcripts of 239 (1.21%) genes were upregulated and the transcripts of 794 (4.01%) genes were downregulated (**Figures 4A, B**). Down-regulated gene ontology (GO) enrichment analysis demonstrated a significant defense response to other organisms and G-protein coupled receptor activity, as well as granulocyte migration/chemotaxis (**Figures 4C, D**). These were associated with the regulation of cell chemotaxis and the cytokine-mediated signaling pathway (**Figure 4D**). Additionally, KEGG pathway analysis demonstrated that the IL-17 signaling pathway, the PI3K-Akt signaling pathway, and the Cytokine-cytokine receptor interaction were all downregulated (**Figure 4E**). The data of RNA-seq indicates that the deletion of the Fpr2 receptor down-regulates many genes related to chemokine and chemokine receptors, which is related to hosting resistance to infection. The 17 infection- and acute-inflammation-related genes initially identified in our RNA-seq experiment were subsequently verified by qPCR (**Figure 4F**). Our results demonstrated that Fpr2 deletion significantly down-regulated the Saa3 expression, which is an important inflammation-related factor during the acute infection phase. Saa can induce several kinds of cytokines/chemokine (17). The classical chemokine CXCL1/2 of neutrophils and its receptor CXCR2 also decreased significantly in Fpr2^-/-^ mice. Another gene closely related to the host control of GBS infection, Il1b (10), was also significantly down-regulated in Fpr2^-/-^ mice. Other factors related to neutrophil chemotaxis and activation of host inflammation, including *S100a8* (18), *TREM1* (19), and the complement receptors *C5ar1* (20, 21) and *ITGAM* (22), were significantly down-regulated in Fpr2^-/-^ mice (**Figure 4F**). **Table 1** provides a list of the primers used in the present study.

**Table 1:** The primers used in this study were listed.

Primer	sequences (5’ to 3’)
**mFpr1-F**	TCCTGATTGCCCTCATTGC
**mFpr1-R**	CCCAGACTGGATGCAGAACA
**mFpr2-F**	TTTACACCACAGGAACCGAAGA
**mFpr2-R**	TAATTTTTTCAGTCCTGCAGCTAACC
**mFpr3-F**	ATGGGCCAGGACTTTCAAGA
**mFpr3-R**	GGCTCTCTGCAGACGAGAAGA
**Saa3-F**	GGGACATGGAGCAGAGACTCA
**Saa3-R**	ACTCCGGCCCCACTCATT
**Egr1-F**	GAACCCCTTTTCAGCCTAGTCA
**Egr1-R**	AGGATGAAGAGGTCGGAGGATT
**Gpr84-F**	CCACGCGTATGGCTCCAT
**Gpr84-R**	ATGGAACCGGCGGAAACT
**S100a8-F**	GGAGTTCCTTGCGATGGTGAT
**S100a8-R**	TCTGCTACTCCTTGTGGCTGTCT
**Itgam-F**	CGTCTGCGCGAAGGAGATAT
**Itgam-R**	CGGCCAGGGCTCTAAAGC
**C5ar1-F**	GAGTGGCCTGGGTCTTAGCA
**C5ar1-R**	TGCCTCCCGGTACACGAA
**Mgl2-F**	AGGTGGAGGCGAGGACTGT
**Mgl2-R**	GGCAGACATCGTCATTCCAA
**CXCL3-F**	GCGCTGTCAGTGCCTGAAC
**CXCL3-R**	CAAGCTCTGGATGGTCTCAAAA
**Msr1-F**	TGGAGGAGAGAATCGAAAGCA
**Msr1-R**	GGAAGCGTTCCGTGTCTATAAGG
**Plcd3-F**	AGCCAAGCAGCACGAACTG
**Plcd3-R**	AGGCGACAACAGATACATCATGA
**Xcr1-F**	TCAGGACTTTGTTTCGCACAA
**Xcr1-R**	CTACCACGACGGTGAAGATGAG
**CXCR2-F**	CCCTCTTTAAGGCCCACATG
**CXCR2-R**	AAGGACGACAGCGAAGATGAC
**CXCR4-F**	TCGGCAATGGATTGGTGAT
**CXCR4-R**	CCGTCATGCTCCTTAGCTTCTT
**CXCL1-F**	TCCCCAAGTAACGGAGAAAGAA
**CXCL1-R**	AGCCAGCGTTCACCAGACA
**CXCL2-F**	TGGGCTGCTGTCCCTCAA
**CXCL2-R**	CCCGGGTGCTGTTTGTTTT
**CXCL12-F**	GCCTCCAAACGCATGCTT
**CXCL12-R**	ATTGGTCCGTCAGGCTACAGA
**IL-1β -F**	CCATGGCACATTCTGTTCAAA
**IL-1β -R**	GCCCATCAGAGGCAAGGA
**GAPDH-F**	CATGGCCTTCCGTGTTCCTA
**GAPDH-R**	GCGGCACGTCAGATCCA

**Table 2:** Clinical score parameters, assessed values.

Clinical score	Value
**0**	Normal
**1**	Mild
**2**	Moderate
**3**	Severe

This table is based on the summary of National Institutes for Food and Drug Control.

0, normal，within the normal range; 1, mild, usually the lesions are easy to identify, but the degree is relatively light; 2, moderate, the lesions are obvious, and there is a significant tendency to aggravate; 3. severe, the lesions are severe, all tissues are cumulatively lifted, occupying organs the vast majority.

Regarding genes related to chemotaxis and neutrophils activation, we detected the CXCL12/CXCR4 axis that mediates neutrophils homing and found a significant decrease in Fpr2^-/-^ mice (**Figure 4F**). These RNA-seq results indicate that Fpr2 deletion could affect neutrophils chemotaxis by altering the expression of CXCL1/2 and other chemokines.

**Figure 5:**
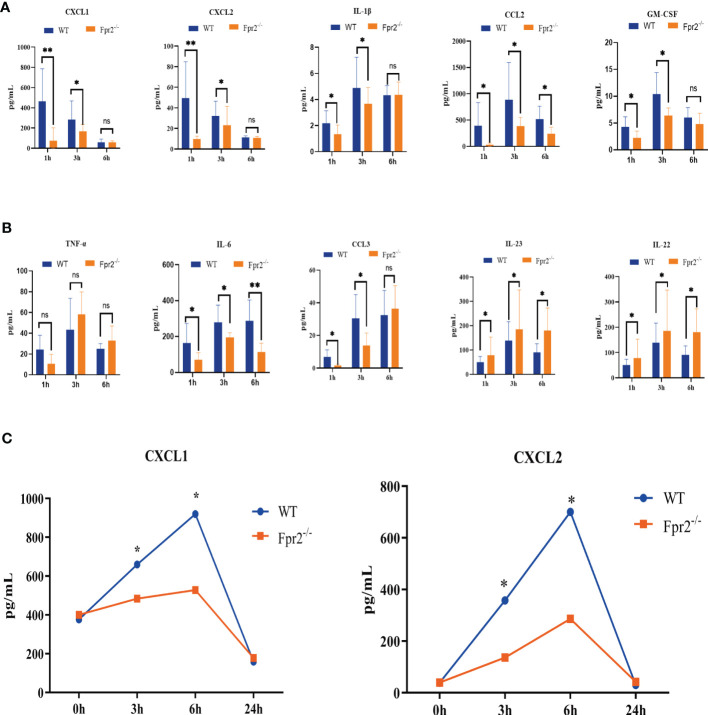
Kinetics of cytokine/chemokine production in peripheral blood after *i.v.* challenge with 2×10^8^ CFU/ml GBS.**(A, B)** CXCL1, CXCL2, IL-1β, CCL2, GM-CSF, TNF-α, IL-6, CCL3, IL-23, and IL-22 protein levels from WT and Fpr2^-/-^ mice were measured at 1h, 3h, and 6h. **(C)** CXCL1/2 concentration in peritoneal lavage fluid after HK-GBS injection. Data are representative of three independent experiments with n = 8 mice per group. Data are shown as mean ± SD. *P < 0.05, **P < 0.01. ns, means non sense.

Neutrophil recruitment in the blood and at infection sites is orchestrated by chemotactic cytokines/chemokines, including GM-CSF, CXCL1/2, TNF-α, and IL-6. Therefore, we next sought to ascertain whether neutrophil chemokine levels decreased in the blood of Fpr2-deficient mice inoculated with live GBS. At early time points, Fpr2^-/-^ mice showed a significant decrease in CXCL1, CXCL2, IL-1β, CCL2, GM-CSF, IL-6, and CCL3 levels at 1 hour and 3 hours after infection, and an increase in IL-22 and IL-23, but there is no change in TNF-α, as compared with WT mice (**Figures 5A, B**). To further confirm the effects of CXCL1/2 on the chemotactic neutrophils of Fpr2^-/-^, we injected HK-GBS *i.p.* and found that the amount of CXCL1/2 in the WT group was significantly higher than in the Fpr2^-/-^ group. Compared with blood CXCL1/2, the content of CXCL1/2 in the abdominal cavity has significant difference between 3 and 6h (**Figure 5C**), which was consistent with changes in neutrophils content observed in the abdominal cavity.

The RNA-seq and luminex results of CXCL1/2 downregulation indicates that Fpr2 is needed for their production and CXCL1/2 play an important role in controlling GBS infections. Meanwhile Fpr2 absence caused an imbalance of neutrophils exudation from the bone marrow and homing.

### Fpr2-PI3K-CXCL1/2 Affects the Chemotaxis of Neutrophils in GBS Infection

**Figure 6:**
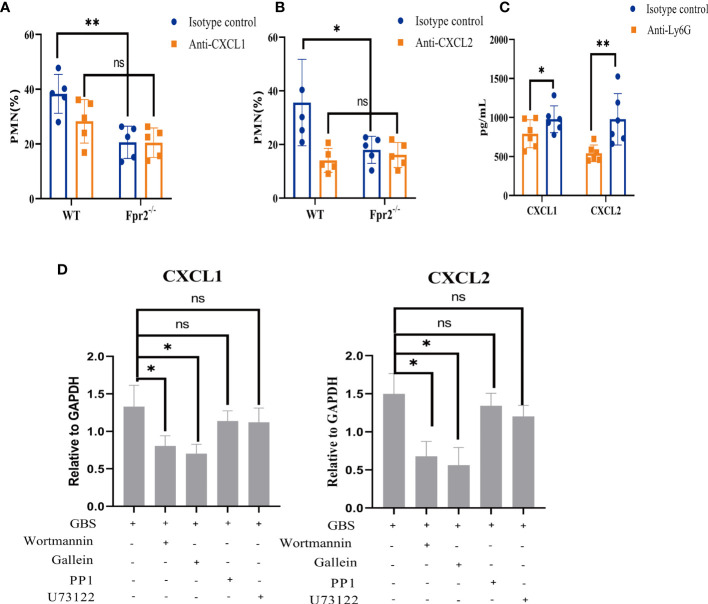
Effects of CXCL1/2 depletion on anti-GBS defenses. **(A, B)** Neutrophils (% of CD45^+^ cells) in peripheral blood of WT and Fpr2^-/-^ mice pretreated with anti-CXCL1 antibody or anti-CXCL2 antibody or the isotype control after *i.v.* challenge with 2×10^8^ CFU/ml of GBS. **(C)** CXCL1 and CXCL2 levels in peripheral blood of WT mice pretreated with anti-Ly6G antibody or the isotype control after *i.v.* challenge with 2×10^8^ CFU/ml of GBS. **(D)** Quantitative analysis of CXCL1 and CXCL2 expression after neutrophils were pretreated with the Gβγ-specific inhibitor Gallein(20μM), the PI3K-specific inhibitor Wortmannin(50μM), the Src-specific inhibitor PP1(20μM), and the PLC specific inhibitor U73122 (50μM) for 1 hour and treated with 2×10^6^ CFU/ml GBS for 1hour (MOI=10:1). Data are representative of three independent experiments with n = 5 mice per group. Data are shown as mean ± SD. *P < 0.05, **P < 0.01, ns means non sense.

The lack of neutrophils is not affected by GBS cytotoxins; meanwhile, chemotaxis detection demonstrates that CXCL1/2 in GBS-infected Fpr2^-/-^ mice slowed the increase of neutrophils. This indicates that, in Fpr2^-/-^ mice, the amount of neutrophils has relationship with the chemokine CXCL1/2. To demonstrate the role of CXCL1 and CXCL2 in mice infected with GBS, 100μg of anti-CXCL1 and anti-CXCL2 antibodies were injected *i.p.* for three hours before the infection. Orbital intravenous injection of 2×10^8^CFU/ml GBS demonstrated that pretreatment with anti-CXCL1/2 eliminated differences in the number of neutrophils in the blood between WT and Fpr2^-/-^mice (**Figures 6A, B**). Compared with CXCL1, blocking CXCL2, a high-affinity ligand of CXCR2, reduced the proportion of neutrophils in the blood (**Figure 6B**). These results demonstrate that for GBS infections, Fpr2 primarily controls neutrophils levels *via* CXCL1/2.

CXCL1/2 can be produced by many cells including neutrophils. To determine which cell produced CXCL1/2, Ly6G antibody and clodronate liposomes were used to block neutrophils and monocytes/macrophages respectively (**Figure 2C**, **Supplementary Figure 1C**). We then used 2×10^8^CFU/ml GBS *via* orbital vein injection. Blood was then obtained for ELISA detection. Our results demonstrated that only blocking neutrophils inhibited CXCL1/2 expression which indicated that CXCL1/2 found in the blood during the early stages of GBS infection is primarily produced by neutrophils (**Figure 6C**, **Supplementary Figure 1D**).

The results of inhibitors showed that GBS may activate Gβγ in Fpr2 heterotrimer and PI3K pathway to produce CXCL1/2 (**Figure 6D**). The activation of Fpr2-PI3K, the release of CXCL1/2, and the subsequent chemotaxis of neutrophils all played an important role in host defense to GBS infections.

### Deletion of the Fpr2 Receptor Affects the Bactericidal Function of Neutrophils

**Figure 7:**
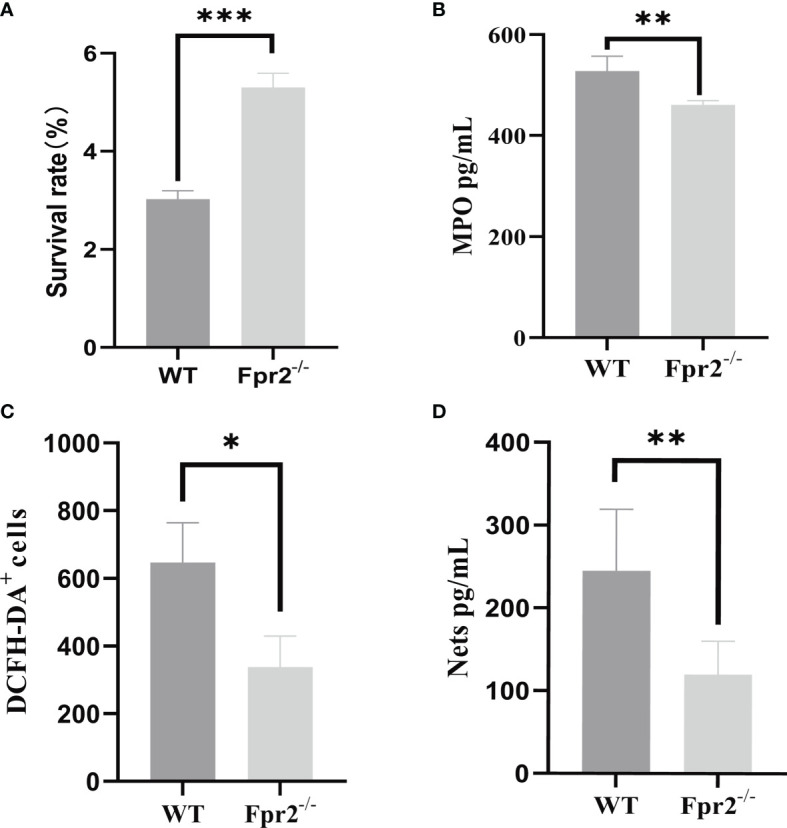
GBS killing, MPO, NETs and ROS production by peripheral blood. **(A)** GBS killing capacity of peripheral blood. Mouse peripheral blood were incubated with 2×10^6^ GBS/ml for 1 h at 37℃. MPO assay **(B)** of peripheral blood and NETs **(C)** ROS **(D)** detection of neutrophils from WT and Fpr2^-/-^ mice incubated with 2×10^6^ CFU/ml of GBS. Data are representative of three independent experiments with n = 4 mice per group. Data are representative of three independent experiments with n = 4 mice per group. Data are shown as mean ± SD. *P < 0.05, **P < 0.01, ***P < 0.001.

In addition to chemotaxis, Fprs also directly mediate neutrophil phagocytosis (23). Therefore, we examined what was capable of killing GBS, including the production of MPO, ROS, and NETs from WT and Fpr2^-/-^ mice. The peripheral blood of mice was added to GBS in the mid-log phase to induce infection, and the results demonstrated that the killing ability of Fpr2-knockout mice was weakened (**Figure 7A**). We detected the expression levels of MPO in the peripheral blood, the results showed that neutrophils of Fpr2-/- mice produced less MPO. We next detected the expression levels of ROS, and NETs in the peripheral blood neutrophils of the mice three hours after infection. These results demonstrated that the expression levels of these two were significantly reduced in Fpr2^-/-^ mice (**Figures 7B–D**) and that bactericidal functions in peripheral blood decreased in Fpr2 knockout mice. Therefore, the Fpr2 receptor not only affects chemotaxis but also affects other functions, including its ability to kill GBS infections.

## Discussion

This study highlights the importance of Fpr2 in host defense against lethal GBS infections. Like previous studies on *L. monocytogenes* and *E. coli*, we found that loss of the Fpr2 receptor decreased the host’s chemotactic ability to neutrophils and a weakening of the bactericidal effects. The mechanism behind chemotaxis is not directly accomplished by Fpr2 chemotactic neutrophils; Fpr2/CXCL1/2 is involved in neutrophil accumulation to control GBS infection. However, the mouse Fpr2 is just a human analog; observations in mouse models should be interpreted with caution when applying these conclusions to humans.

Fpr2 is a chemotactic receptor, and its deletion will affect the innate immunity of the host. The effects of Fpr2^-/-^ mice on host anti-infection has been studied in *S. pneumoniae* (24, 25), *S. aureus* (26, 27), *S. suis* (28), *L. monocytogenes* (13), and *E. coli* (6). All of these studies have found that loss of the Fpr2 receptor increases the bacterial load and mortality of the host, though the specific mechanism is different for different pathogens. In the meningitis model (caused by *S. suis* and *S. pneumoniae* infections) and pneumonia (caused by *S. pneumoniae* infections), Fpr2 deletion increased the infiltration of neutrophils in CNS and lung, however, the infiltrating neutrophils were in an “inflamed” but “incompetent” state, resulting in a higher fatality rate (24, 25, 28). For *S. aureus*, *L. monocytogenes*, and *E. coli*, Fpr2^-/-^ mice did lead to reduced neutrophils infiltration, uncontrolled bacterial growth, and impaired neutrophils function at the infected site. This is likely due to a lack of inflammatory responses, leading to a decrease in the survival rate of mice (6, 13, 29). But in either case, lack of Fprs does damage neutrophils function. This study assessed GBS infections and found that regardless of whether the injection method was *i.v.* or *i.p.*, Fpr2 activation was very important for neutrophils recruitment, host control of the GBS infection, and improvement of the survival rate.

Studies assessing *L. monocytogenes* and *E. coli* infections demonstrated that Fpr2 is a direct chemotactic neutrophils factor during early immune responses. However, for GBS, after excluding the effects of hemolysin βH/C, the host chemotactic effect on neutrophils is not through Fpr2 directly, but indirectly by activating Fpr2 to produce chemokine CXCL1/2. Neutrophils comes from bone marrow, and its migration into the blood is based on the regulation of G-CSF in bone marrow exudation-related CXCL1/2/CXCR2 and homing-related CXCL12/CXCR4 (30, 31). The results of RNA-seq and ELISA analyses demonstrated that Fpr2 deletion simultaneously down-regulated CXCL1/2 and its receptor CXCR2, and CXCL12, and its receptor CXCR4, but did not affect G-CSF expression.

While differences in neutrophils in the blood between WT and Fpr2^-/-^mice could not be due to different G-CSF expressions in the two mice groups, our results demonstrate that Fpr2 loss disturbed the balance between CXCL1/2/CXCR2 and CXCL12/CXCR4 and unbalancing the exudation and homing of neutrophils from bone marrow. However, this was not necessarily the reason for the difference in neutrophils between the two mice groups.

After detecting the inflammatory factors, we found that both IL-1β and CXCL1/2 decreased in Fpr2^-/-^ mice. Previous studies by *C. Biondo*, have demonstrated that the loss of IL-1β or its receptors can reduce the production of CXCL1/2, thus reducing chemotaxis to neutrophils and affecting the host’s ability to control GBS infections (10, 32). As such, decreases in neutrophils caused by Fpr2 deletion are likely due to decreases in IL-1β expression, which affects CXCL1/2 production. There was no difference in the production level of TNF-α between the Fpr2^-/-^ and WT groups in the first six hours after infection.

The cells that can produce CXCL1/2 include neutrophils, monocytes, and endothelium and etc. In this study, we found that the production of CXCL1/2 in the blood primarily came from neutrophils. Previous studies on GBS infections found that CXCL1/2 quickly increased due to GBS infections, which typically peak approximately 3-6 hours after infection (10, 32). However, the CXCL1/2 release as a result of *L. monocytogenes* and *E. coli* infections was much slower. CXCL1/2 in *L. monocytogenes* infection model was detected only at 4-8 hours, while CXCL1 peaked at 24 hours (13). For *E. coli*, the production of CXCL1 and CXCL2 was minimal at 3 h and reached peak at 8 h (6). These two bacteria chemotactic neutrophils directly through Fpr2, meaning late CXCL1/2 production does not affect the chemotaxis of early neutrophils. Moreover, PAMP, the end-target of neutrophils, exhibits faster and more accurate chemotaxis to neutrophils compared with CXCL1/2. This enhances the interaction of the pattern recognition receptor (PRR) with other PAMPs and enhances the antibacterial response of the host. However, for GBS infection, repeated experiments have demonstrated that if CXCL1/2 decreases, neutrophils recruitment will significantly decrease. Similarly, CXCL1 and CXCL2 quickly increased in the early stages of GBS infection, while a significant difference in these two factors in WT and Fpr2^-/-^ can be detected at 1 hour and 3 hours for both *i.v.* and *i.p.* infection route, which is consistent with that of the neutrophils increase. The rapid release of CXCL1/2 is caused by GBS infections, which could be because mature human neutrophils contain small amounts of interleukin-8 (CXCL1/2 compartment) (33), which significantly increases upon proinflammatory activation.

When we previously studied the release of primary granule HBPs stimulated by *S. aureus* PSM, we found that the particle movement of neutrophils was related to the activation of PI3K and cytoskeleton rearrangement, which is a key signal molecule downstream of Fpr2 (34). IL-8 is present in resting peripheral blood neutrophils and is stored in an easily mobilized organelle (33). We found that the key signal molecules involved in CXCL1/2 release are also related to Gβγ-PI3K. Therefore, CXCL1/2 is likely to be initially released through the movement of particles.

Host neutrophils and macrophages play a principal role in innate immunity: the generation of reactive oxygen species and other antimicrobial substances within the phagolysosome. Studies assessing the control of the *S. aureus* infection by Fprs demonstrate that Fprs activation can enhance the ability of the host to kill bacteria *via* increased complement receptor and FCγR expression on the surface of the neutrophils (23). NETs, an extracellular extrusions of a dense, fibrous matrix comprised of DNA and antimicrobial proteins, is part of a host defense mechanism that sequesters pathogens and slows their invasive progression (35, 36), which is also one of the important methods to resist GBS infection. The absence of Fpr2 influences the ability of neutrophils to kill GBS, which is accompanied by a decrease in the production of ROS, NETs, and MPO release. This is consistent with the effect of Fprs deletion on *S. pneumoniae* and *S. suis*: although a large number of neutrophils gather at the infection site, many bacteria survive due to a reduction in their antibacterial properties. This results in an imbalance of the pro-inflammatory/anti-inflammatory response.

Fpr1 primarily recognizes peptides with the N-terminal formylated methionine, and Fpr2 has been shown to also recognize several non-formylated peptide ligands. The results on *S. aureus* PSM demonstrated that Fpr2 tends to recognize that peptides with longer binding lengths, amphiphilic structures, and α helix. Therefore, the Fpr2 ligands in GBS require further study.

From this study, we found that Fpr2 receptors affect host resistance to GBS infections in many ways, including neutrophils chemotaxis and bactericidal function. For chemotaxis, we proposed that Fpr2 does not directly chemotactic neutrophils, but releases CXCL1/2 chemotactic neutrophils *via* receptor activation. As for the function, we found that the absence of Fpr2 affected the bactericidal ability of neutrophils. Therefore, Fpr2 plays an important role in host resistance to GBS infections.

## Data Availability Statement

The datasets presented in this study can be found in online repositories. The names of the repository/repositories and accession number(s) can be found below: https://www.ncbi.nlm.nih.gov/, PRJNA767365.

## Ethics Statement

The animal study was reviewed and approved by Animal center of the Academy of Military Medical SCiences.

## Author Contributions

ZS performed the whole experiment and analysis. ZS wrote the first draft of the manuscript. HJ and YJ designed the study and revised the manuscript. All authors contributed to manuscript revision, and read and approved the submitted version.

## Funding

This work was supported by grants from the National Natural Science Foundation of China (82002116), the State Key Laboratory of Pathogen and Biosecurity (SKLPBS2119). The funders had no role in study design, data collection and analysis, decision to publish, or preparation of the manuscript.

## Conflict of Interest

The authors declare that the research was conducted in the absence of any commercial or financial relationships that could be construed as a potential conflict of interest.

## Publisher’s Note

All claims expressed in this article are solely those of the authors and do not necessarily represent those of their affiliated organizations, or those of the publisher, the editors and the reviewers. Any product that may be evaluated in this article, or claim that may be made by its manufacturer, is not guaranteed or endorsed by the publisher.

## Supplementary Material

Supplementary Figure 1
**(A)** Quantitative analysis of mFPR1, mFPR2, mFPR3 in peripheral blood after *i.v.* challenge with 2×10^8^ CFU/ml of GBS in WT mice. **(B)** Survival of WT mice i.v. challenge with 2×10^8^ CFU/ml of GBS after treated with Boc-2 (600 ng/kg). **(C)** Evaluation of clodronate liposomes on monocyte/macrophage depletion. WT and Fpr2^-/-^ mice were injected *i.p*. with 200mg/mouse of clodronate liposomes and its control respectively. 24h later the mice were infection *via* orbital vein with GBS and 3h after infection, blood was collected for analysis of monocyte number by flow cytometry. **(D)** Effect of monocyte/macrophage depletion on CXCL1/2 production. WT and Fpr2^-/-^ mice were injected *i.p.* with 1mg/0.2 ml of clodronate liposomes and its control respectively. 24h later the mice were infection *via* orbital vein with GBS and 3h after infection, blood was collected and serum were isolated for analysis of the production of CXCL1/2 by ELISA. ns, not significant. **(E, F)** DPPC block effect evaluation on βH/C. Mouse whole blood and isolated neutrophils was used to test the DPPC on βH/C. Blood or isolated neutrophils from WT mouse were exposed to the GBS strains at MOI of 10:1 with or without DPPC (150, 250, 500μg/ml) for 1h. The mixture was centrifugated and the supernatant was collected for hemolysis(E) and LDH(F) determination by colorimetric assay.**P* < 0.05, ***P* < 0.01, ****P* < 0.001.
