# Supplementary figures and images for: Fpr2/CXCL1/2 Controls Rapid Neutrophil Infiltration to Inhibit Streptococcus agalactiae Infection
Source: Front Immunol. 2021 Nov 24;12:786602. doi: 10.3389/fimmu.2021.786602 (PMC8652123; doi:10.3389/fimmu.2021.786602)

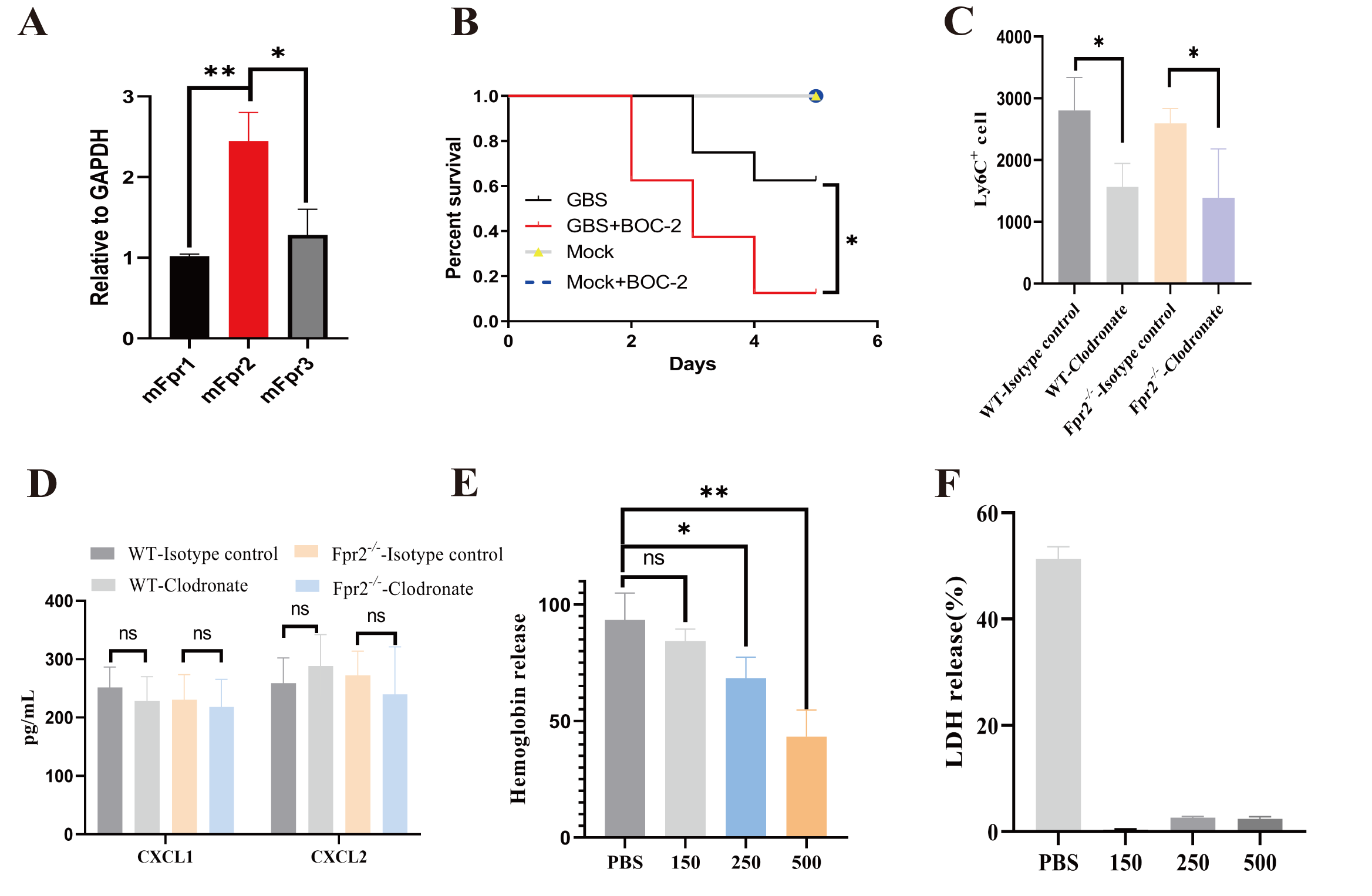

Supplement: Supplementary Figure 1 — (A) Quantitative analysis of mFPR1, mFPR2, mFPR3 in peripheral blood after i.v. challenge with 2×10^8^ CFU/ml of GBS in WT mice. (B) Survival of WT mice i.v. challenge with 2×10^8^ CFU/ml of GBS after treated with Boc-2 (600 ng/kg). (C) Evaluation of clodronate liposomes on monocyte/macrophage depletion. WT and Fpr2-/- mice were injected i.p. with 200mg/mouse of clodronate liposomes and its control respectively. 24h later the mice were infection via orbital vein with GBS and 3h after infection, blood was collected for analysis of monocyte number by flow cytometry. (D) Effect of monocyte/macrophage depletion on CXCL1/2 production. WT and Fpr2-/- mice were injected i.p. with 1mg/0.2 ml of clodronate liposomes and its control respectively. 24h later the mice were infection via orbital vein with GBS and 3h after infection, blood was collected and serum were isolated for analysis of the production of CXCL1/2 by ELISA. ns, not significant. (E, F) DPPC block effect evaluation on βH/C. Mouse whole blood and isolated neutrophils was used to test the DPPC on βH/C. Blood or isolated neutrophils from WT mouse were exposed to the GBS strains at MOI of 10:1 with or without DPPC (150, 250, 500μg/ml) for 1h. The mixture was centrifugated and the supernatant was collected for hemolysis(E) and LDH(F) determination by colorimetric assay.*P < 0.05, **P < 0.01, ***P < 0.001. [file Image_1.tif]
